# Evaluation of Clinical and Pathological Outcomes of Medical Thoracoscopy in Southern Iran: Six Years’ Experience

**DOI:** 10.1016/j.opresp.2026.100670

**Published:** 2026-09-03

**Authors:** Parnian Mansouri, Mohammad Javad Fallahi, Seyed Masoom Masoompour, Navid Omidifar

**Affiliations:** a Student Research Committee, Shiraz University of Medical Sciences, Shiraz, Iran; b Thoracic and Vascular Surgery Research Centre, Department of Internal Medicine, Shiraz University of Medical Sciences, Shiraz, Iran; c Pulmonary and Critical Care Division, Department of Internal Medicine, Shiraz University of Medical Sciences, Shiraz, Iran; d Department of Pathology, Shiraz University of Medical Sciences, Shiraz, Iran

**Keywords:** Medical thoracoscopy, Exudative pleural effusion, Pathological diagnosis, Toracoscopia médica, Derrame pleural exudativo, Diagnóstico anatomopatológico

## Abstract

**Introduction:**

Medical thoracoscopy (MT) is an important diagnostic and therapeutic procedure for pleural diseases, particularly unexplained exudative pleural effusions. Although widely used internationally, data describing MT outcomes from southern Iran remain limited.

**Material and methods:**

This retrospective descriptive study evaluated 93 patients who underwent MT at a large tertiary referral hospital in southern Iran between 2017 and 2023. Data regarding patient characteristics, anesthesia methods, procedural details, complications, and pathological findings were collected and analyzed using descriptive statistics.

**Results:**

The study included 93 patients (59% male and 41% female) with a mean age of 60.7 ± 15.07 years. Conscious sedation was the most commonly used anesthesia method (72%). Pathological examination revealed malignant pleural involvement in 63 patients, including newly diagnosed primary lung malignancies and metastatic pleural disease. Thoracoscopic talc poudrage was performed in 17 patients with recurrent symptomatic pleural effusions. Non-specific inflammatory findings were identified in 27 patients, and three cases of granulomatous inflammation, including tuberculosis, were also observed. Complications occurred in 13 patients, mainly including chest pain, mild intraprocedural bleeding, and respiratory complications. Five patients died during hospitalization; however, none of these deaths were attributed to procedure-related complications.

**Conclusion:**

In this study, MT was associated with a high proportion of definitive pathological diagnoses and a low frequency of severe procedure-related complications. These findings support the utility of MT in the evaluation of selected patients with complex pleural diseases, particularly unexplained exudative pleural effusions. Further studies focusing on patient selection, therapeutic outcomes, and long-term follow-up are warranted.

## Graphical abstract

**Figure:**
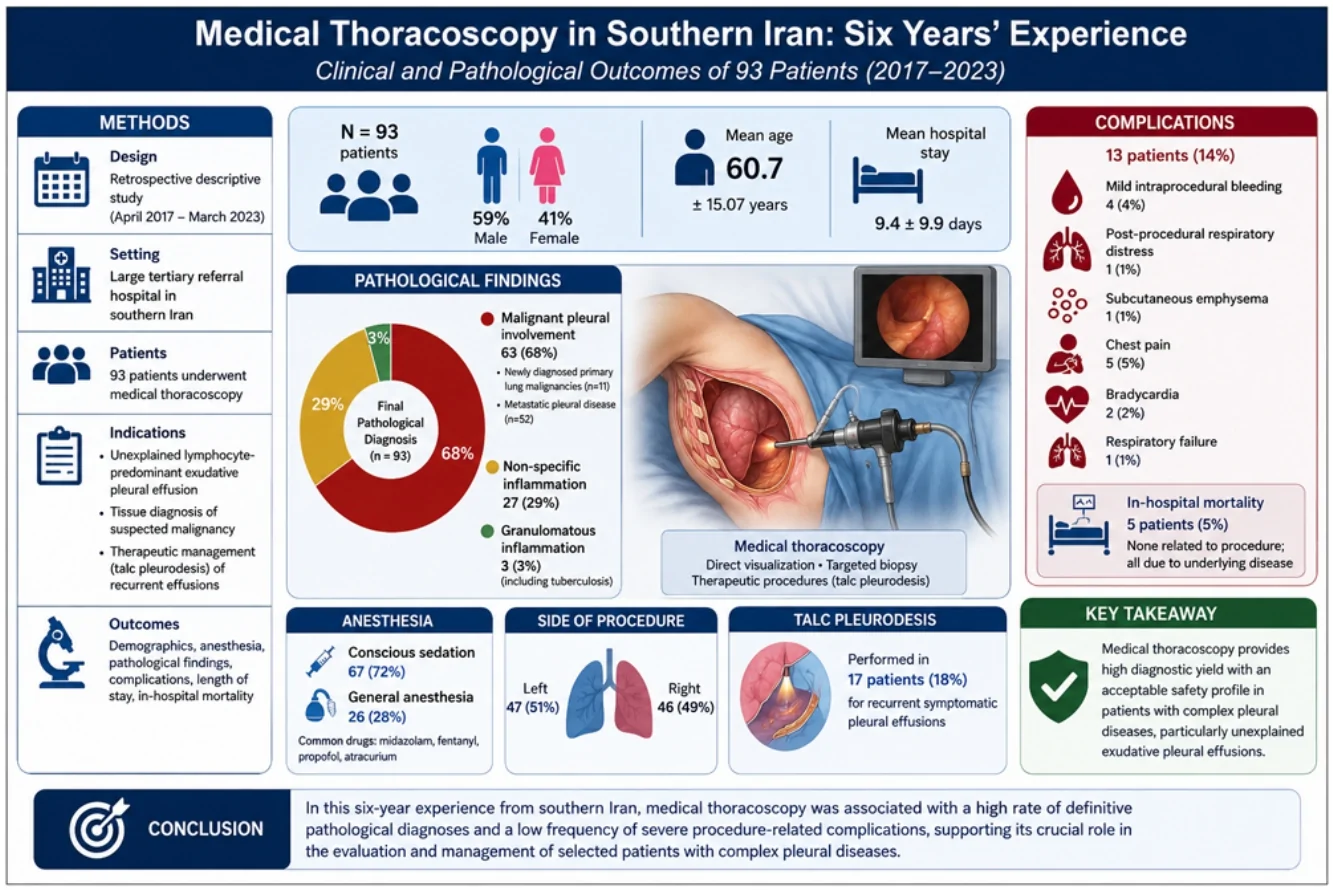

## Abstract (es)

**Introducción:**

La toracoscopia médica (TM) es un procedimiento diagnóstico y terapéutico importante para las enfermedades pleurales, especialmente para los derrames pleurales exudativos de etiología no esclarecida. Aunque su uso está ampliamente extendido a nivel internacional, los datos sobre los resultados de la TM en el sur de Irán siguen siendo limitados.

**Material y métodos:**

En este estudio descriptivo retrospectivo se evaluaron 93 pacientes sometidos a TM en un hospital terciario de referencia en el sur de Irán entre 2017 y 2023. Se recopilaron datos sobre las características de los pacientes, métodos anestésicos, detalles del procedimiento, complicaciones y hallazgos anatomopatológicos, y se analizaron mediante estadística descriptiva.

**Resultados:**

El estudio incluyó 93 pacientes (59% hombres y 41% mujeres), con una edad media de 60,7 ± 15,07 años. La sedación consciente fue el método anestésico más utilizado (72%). El estudio anatomopatológico identificó afectación pleural maligna en 63 pacientes, incluyendo neoplasias pulmonares primarias recién diagnosticadas y enfermedad pleural metastásica. La pleurodesis con talco mediante toracoscopia se realizó en 17 pacientes con derrames pleurales sintomáticos recurrentes. Se identificaron hallazgos inflamatorios inespecíficos en 27 pacientes y tres casos de inflamación granulomatosa, incluida la tuberculosis. Se registraron complicaciones en 13 pacientes, principalmente dolor torácico, sangrado intraprocedimiento leve y complicaciones respiratorias. Cinco pacientes fallecieron durante la hospitalización; sin embargo, ninguna de estas muertes fue atribuida a complicaciones relacionadas con el procedimiento.

**Conclusión:**

En este estudio, la TM se asoció con una alta proporción de diagnósticos anatomopatológicos definitivos y una baja frecuencia de complicaciones graves relacionadas con el procedimiento. Estos hallazgos respaldan la utilidad de la TM en la evaluación de pacientes seleccionados con enfermedades pleurales complejas, particularmente derrames pleurales exudativos de causa no esclarecida. Se requieren estudios adicionales centrados en la selección de pacientes, los resultados terapéuticos y el seguimiento a largo plazo.

## Introduction

Accurate evaluation of pleural effusions, a common clinical finding in both benign and malignant conditions, is essential for optimal patient management. Traditionally, imaging techniques such as chest X-ray, ultrasonography, and chest CT scan, in conjunction with fluid aspiration and analysis, represent the first line of diagnostic evaluation.^1^ While these non-invasive approaches provide useful information, they may be insufficient to distinguish among different causes of pleural effusion, particularly in complex or ambiguous cases. In such situations, when thoracentesis and imaging yield inconclusive results, pleural biopsy (closed or thoracoscopic) becomes an important tool for further investigation.^2^ Over time, thoracoscopy has been increasingly utilized for the evaluation of undiagnosed pleural effusions, pneumothorax, staging of lung cancer with pleural involvement, and primary pleural malignancies.^3^ Beyond diagnosis, thoracoscopy is also an important tool for evaluating conditions such as mesothelioma, lung cancer, diffuse pleural disorders, and tuberculous pleurisy, while providing therapeutic options including chemical or mechanical pleurodesis.^4^, ^5^, ^6^

This procedure can be performed using either video-assisted thoracoscopic surgery (VATS) or medical thoracoscopy (MT), with pulmonologists frequently opting for the latter because of its minimally invasive nature and favorable safety profile.^7^ Although alternative methods, such as closed pleural biopsy with or without imaging guidance, are available, MT has become an important approach for investigating exudative pleural effusions, offering a high diagnostic yield in appropriately selected patients.^8^

In Iran, published data evaluating MT outcomes remain limited. Studies conducted at institutions such as Masih Daneshvari and Kasra Hospitals have demonstrated favorable diagnostic yield and safety profiles, with low rates of procedure-related mortality.^9^, ^10^ Few studies have evaluated the outcomes of MT in southern Iran.^11^ Although MT is an established procedure for the evaluation of pleural disease, real-world data describing clinical characteristics, pathological findings, procedural outcomes, and complications remain limited in some regions. Therefore, this study aimed to describe the clinical characteristics, pathological findings, procedural outcomes, and complications of patients undergoing medical thoracoscopy at a tertiary referral center in southern Iran over a six-year period.

## Material and methods

### Study design

This retrospective descriptive study included all patients who underwent medical thoracoscopy at a large tertiary referral hospital in southern Iran between April 2017 and March 2023. The objective of this study was to describe the clinical characteristics, pathological findings, procedural outcomes, and complications associated with medical thoracoscopy.

### Study population

The study included all inpatient and outpatient cases that underwent medical thoracoscopy during the study period, with no exclusion criteria applied. The main indications for medical thoracoscopy were unexplained lymphocyte-predominant exudative pleural effusions despite initial diagnostic evaluation. In patients with known or suspected malignancy, MT was performed either for obtaining sufficient tissue for definitive histological subtyping and comprehensive molecular profiling, specifically to identify driver mutations and actionable therapeutic targets or for therapeutic management of recurrent symptomatic pleural effusions, including talc pleurodesis when lung re-expansion was achievable and pleurodesis was considered clinically appropriate. Before each procedure, pleural ultrasonography was performed to determine the optimal site of entry. All procedures were performed by an experienced pulmonologist using a Karl Storz® rigid thoracoscope (telescope number: 26038AA) with the assistance of pulmonary fellows.

### Data collection

Pre-procedural suspected/definite diagnosis were established based on operation notes and archived patient records. Demographic data, including age and sex, as well as baseline diagnoses and indications for the procedure, were collected. Existing comorbidities and underlying diseases were also recorded. Additional procedural data included the side of the chest, type of anesthesia or sedation, related medications, and duration of hospital stay. Procedure-related complications were documented based on radiologic findings and clinical reports, and in-hospital mortality was recorded. Final pathological diagnoses were obtained through review of pathology reports. Previous pleural fluid cytology results and long-term clinical outcomes were not consistently available in medical records and therefore were not included in the analysis.

### Data analysis

Variables were summarized using descriptive statistics, including absolute and relative frequencies for categorical variables and mean ± standard deviation for continuous variables. As this study was primarily descriptive in nature, no inferential statistical tests were performed.

## Results

A total of 93 patients underwent medical thoracoscopy at a large tertiary referral hospital in southern Iran between 2017 and 2023. The study comprised 38 females (41%) and 55 males (59%), with a mean age of 60.7 ± 15.07 years. Thoracoscopic talc poudrage was performed in 17 patients (8 men and 9 women, with a mean age of 62.7 ± 13.45 years) with recurrent symptomatic pleural effusions requiring therapeutic management. The remaining patients with malignant pleural involvement either underwent MT primarily for diagnostic purposes or were not considered suitable candidates for pleurodesis due to their clinical status or disease characteristics. The average duration of hospital admission was 9.4 ± 9.9 days (Table 1).

**Table 1:** Patient's demographic and basic data.

Variable	Sub-type	No (%), mean ± SD
Gender	Women	38 (41%)
	Men	55 (59%)
Age (years)		60.7 ± 15.07
Type of anesthesia	Sedation	67 (72%)
	General	26 (28%)
Hospital stay (days)		9.4 ± 9.9
Chest wall side	Left	47 (50.5%)
	Right	46(49.5%)

Conscious sedation was the most common anesthesia method, used in 67 cases (72%), followed by general anesthesia in 26 cases (28%), mainly to facilitate pleurodesis. The most frequently used anesthetic medications included fentanyl, midazolam, propofol, and atracurium. The most common drug combinations were midazolam with fentanyl, used in 35 cases, and a combination of midazolam, fentanyl, sodium thiopental (STP), and atracurium, used in 13 cases.

Complications were observed in 13 patients (14%), while 80 patients (86%) experienced no procedure-related complications. Documented complications included mild intraprocedural bleeding in 4 cases, post-procedural respiratory distress in 1 case, subcutaneous emphysema in 1 case, chest pain in 5 cases, bradycardia in 2 cases, and respiratory failure in 1 case (Table 2). One patient experienced multiple complications, including mild bleeding, pain, and bradycardia. The procedures were almost equally distributed between the left chest wall (47 cases) and the right chest wall (46 cases) (Table 1).

**Table 2:** Patient's underlying diseases.

*Malignancy*	
Lung cancer	18/93 (19.4%)
Breast cancer	6/93 (6.5%)
Multiple myeloma	2/93 (2.1%)
Gastric cancer	2/93 (2.1%)
Esophageal cancer	1/93 (1.1%)
Ovarian cancer	1/93 (1.1%)
Unknown metastatic cancer	1/93 (1.1%)
Synovial sarcoma	1/93 (1.1%)
Renal cell carcinoma	1/93 (1.1%)
Prostate cancer	1/93 (1.1%)
Colon cancer	1/93 (1.1%)
ALL with stem cell transplant	1/93 (1.1%)

*Mass*	
Lung	4/93 (4.2%)
Adrenal	1/93 (1.1%)

*IHD*	11/93 (11.8%)
*COPD*	7/93 (6.7%)
*Asthma*	4/93 (4.2%)
*CHF*	3/93 (3.2%)
*Hepatitis*	2/93 (2.1%)
*ESRD*	2/93 (2.1%)
*Empyema*	1/93 (1.1%)
*RA*	1/93 (1.1%)

*Total*	72/93 (77.4%)

ALL: acute lymphoblastic leukemia; IHD: ischemic heart disease; COPD: chronic obstructive pulmonary disease; CHF: congestive heart failure; ESRD: end-stage renal disease; RA: rheumatoid arteritis.

Seventy-two patients had underlying diseases, with malignancies present in 36 cases. The most frequent primary malignancies were lung cancer and breast cancer. Other malignancies included multiple myeloma and gastric cancer in two cases each, as well as individual cases of esophageal cancer, ovarian cancer, synovial sarcoma, renal cell carcinoma, prostate cancer, colon cancer, acute lymphoblastic leukemia (ALL) with stem cell transplantation, and metastatic disease from an unknown primary source (Table 2).

Pathological examination revealed malignant pleural involvement in 63 patients. Among these, 11 cases represented newly diagnosed primary lung malignancies, including 7 adenocarcinomas, 2 tumors of undefined type, 1 non-small cell lung cancer (NSCLC), and 1 small cell lung cancer (SCLC). Malignant pleural involvement associated with previously diagnosed and/or treated lung cancer was identified in 27 cases, most commonly adenocarcinoma subtype (Table 3).

**Table 3:** Patient's final pathology reports.

Final pathology report	Sub-type	No (%)
*Malignant pleural effusion*		63/93 (67.6%)
Primary lung cancer	Adenocarcinoma	7/11 (63.6%)
	Undefined type	2/11 (18.2%)
	Non-small cell lung cancer	1/11 (9.1%)
	Small cell lung cancer	1/11 (9.1%)
	Total	11/63 (17.5%)

Lung metastasis	Adenocarcinoma	17/27 (62.9%)
	Undefined type	6/27 (22.2%)
	Squamous cell cancer	3/27 (11.1%)
	Small cell lung cancer	1/27 (3.8%)
	Total	27/63 (42.8%)

Malignant mesothelioma	Total	7/63 (11.1%)

Extra-pulmonary malignancy	Breast cancer	6/18 (33%)
	Multiple myeloma	2/18 (11.1%)
	Unknown metastatic cancer	2/18 (11.1%)
	Esophageal cancer	1/18 (5.6%)
	Ovarian cancer	1/18 (5.6%)
	Soft tissue sarcoma	1/18 (5.6%)
	Synovial sarcoma	1/18 (5.6%)
	Renal cell carcinoma	1/18 (5.6%)
	Colon cancer	1/18 (5.6%)
	Thyroid cancer	1/18 (5.6%)
	Epithelioid hemangio-endothelioma	1/18 (5.6%)
	Total	18/63 (28.6%)

*Non-specific inflammation*		27/93 (29.1%)
With underlying disease	Lung masses	4/17 (23.5%)
	Heart failure	2/17 (11.8%)
	Gastric cancer	2/17 (11.8%)
	End stage renal disease	2/17 (11.8%)
	Lung cancer	1/17 (5.9%)
	Prostate cancer	1/17 (5.9%)
	Adrenal mass	1/17 (5.9%)
	Empyema	1/17 (5.9%)
	Rheumatoid arthritis	1/17 (5.9%)
	Chronic obstructive pulmonary disease	1/17 (5.9%)
	Acute lymphoblastic leukemia	1/17 (5.9%)
	Total	17/27 (63%)

Without underlying disease		10/27 (37%)

*Granulomatous inflammation*		3/93 (3.3%)

Three patients were diagnosed with granulomatous inflammation. Two of these patients were initially suspected of having tuberculosis (TB) and were ultimately diagnosed with TB. The third patient, who had chronic obstructive pulmonary disease (COPD) and an unexplained pleural effusion, demonstrated caseating granuloma on pathological examination. Although acid-fast bacilli testing was negative, the patient was treated for tuberculous pleuritis.

Five patients died during hospitalization; however, none of the deaths were attributed to procedure-related complications. All deaths occurred in the context of advanced underlying diseases and severe comorbid conditions (Table 2).

## Discussion

Medical thoracoscopy plays a critical role in the diagnostic evaluation and therapeutic management of pleural diseases.^12^, ^13^ In our study, it demonstrated high clinical value by establishing definitive pathological diagnoses in a substantial number of patients, particularly those with malignant pleural disease, while also providing therapeutic benefit through pleuroscopic talc pleurodesis in selected symptomatic cases. These findings highlight the practical importance of medical thoracoscopy in pleural practice, especially when less invasive diagnostic approaches fail to provide a definitive diagnosis.^14^, ^15^

Our results are consistent with contemporary pleural disease guidelines. The British Thoracic Society guideline recommends thoracoscopic or image-guided pleural biopsy for undiagnosed pleural effusion after initial clinical evaluation and pleural aspiration, emphasizing targeted tissue sampling in unresolved cases.^16^ Recent European pleural literature likewise supports medical thoracoscopy as a high-yield procedure for undiagnosed exudative effusions, particularly when malignancy or tuberculosis is suspected, because it allows direct visualization of the pleura and targeted biopsy.^17^

In our investigation, the diagnostic value of thoracoscopy was most evident in malignant pleural disease, where malignant pleural involvement was identified in 63 patients, including newly diagnosed primary lung malignancies and metastatic pleural disease. This supports the current preference for obtaining pleural tissue when routine workup remains inconclusive, since thoracoscopy can provide adequate samples for histopathologic confirmation and, when needed, further oncologic characterization. The same rationale is reflected in current BTS recommendations and recent studies demonstrating high diagnostic yields for malignant pleural effusions.^16^

The relatively low number of cases of tuberculous pleuritis in our study may be explained by the diagnostic pathway used in our center. In several patients, tuberculosis was diagnosed before thoracoscopy through sputum acid-fast bacilli testing, bronchoscopy samples, or pleural adenosine deaminase (ADA) measurement, which may have reduced the number of tuberculous cases entering the thoracoscopic pathway. Therefore, the distribution of diagnoses should be interpreted in the context of local referral patterns and pre-thoracoscopy diagnostic evaluations rather than as a direct indicator of disease prevalence.

Non-specific pleuritis remains one of the most important limitations of medical thoracoscopy because a benign-appearing biopsy may still represent sampling error or an early manifestation of pleural malignancy. Previous series have reported later malignant diagnoses in approximately 5–12% of NSP cases, with mesothelioma accounting for most false-negative outcomes, and pleural effusion recurrence has been associated with an increased likelihood of missed malignancy.^18^ Although systematic follow-up was not available in current investigation, one patient initially diagnosed with NSP developed recurrent pleural effusion after about 1.5 years and was subsequently confirmed to have malignant mesothelioma by VATS performed by a thoracic surgeon. Therefore, NSP should not be regarded as a final diagnosis in patients with ongoing or recurrent effusion, and close clinical reassessment remains warranted.^19^

Medical thoracoscopy can be performed under local anesthesia with sedation or general anesthesia, depending on the patient's condition and procedural objectives. The choice of anesthesia is important for minimizing discomfort and reducing procedural risks.^20^, ^21^ In our study, the most commonly used anesthetic medications were fentanyl, midazolam, propofol, and atracurium. Fentanyl and midazolam, in particular, are frequently used in procedural sedation because of their synergistic analgesic and anxiolytic effects, which have been supported by previous studies.^22^, ^23^ Our investigation has similarly demonstrated that patients undergoing MT with local anesthesia and sedation tolerated the procedure well, consistent with previous reports regarding the feasibility of this approach.^24^

The safety profile of medical thoracoscopy in our experience was acceptable, with most patients experiencing no complications and adverse events occurring in a small proportion of cases. This is consistent with recent reports describing thoracoscopy as a generally safe procedure when performed in experienced centers and with appropriate patient selection.^25^ One patient experienced a combination of complications, including bradycardia, mild bleeding, and pain; however, these events were not life-threatening. The occurrence of these adverse events highlights the need for careful peri-procedural monitoring, particularly in patients with advanced malignancy or significant comorbidities.

Prolonged hospitalization in some patients was more likely related to variation in chest tube removal practices and the severity of underlying disease rather than thoracoscopy itself. Likewise, the observed mortality should be interpreted cautiously, as many patients in this study had advanced malignancy and substantial comorbidities at baseline. These patients were referred for evaluation or management of complex pleural diseases, and mortality was related to progression of underlying disease rather than the thoracoscopic procedure itself. This highlights the importance of thorough pre-procedural evaluation to assess patient suitability for MT, particularly among those with significant comorbidities.

## Limitations and conclusion

While our study provided valuable insights into the utility of MT, several limitations exist, including its retrospective design. All procedures were performed by a single experienced operator, which may have reduced inter-operator variability but could also limit the generalizability of our findings. The single-center setting and limited follow-up further restrict the applicability of our results. Additionally, previous pleural fluid cytology results and standardized follow-up data regarding therapeutic outcomes, particularly after talc pleurodesis, were unavailable. Therefore, the therapeutic effectiveness of MT could not be formally assessed. The absence of a comparison group also prevented evaluation of the impact of MT on clinical outcomes. Furthermore, follow-up information for patients diagnosed with non-specific pleuritis was unavailable, limiting assessment of subsequent diagnoses or long-term outcomes.

Future studies should focus on prospective data collection, standardized follow-up, and evaluation of patient selection criteria and therapeutic outcomes in specific clinical scenarios.

In conclusion, our findings provide real-world data on the clinical characteristics, pathological outcomes, and safety profile of MT in a tertiary referral setting. MT was associated with a high proportion of definitive pathological diagnoses and an acceptable rate of procedure-related complications, supporting its role in the evaluation of selected patients with complex pleural diseases.

## Ethics approval and consent to participate

This study was conducted in accordance with the ethical principles outlined in the Declaration of Helsinki. The study protocol was reviewed and approved by the Ethics Committee of Shiraz University of Medical Sciences (SUMS) under the code IR.SUMS.MED.REC.1402.397. Written informed consent was obtained from all participants or their legal guardians prior to the procedures.

## Artificial intelligence involvement

AI was used for language editing and text refinement only; all scientific content was verified and approved by the authors.

## Consent for publication

Not applicable.

## Funding

This research was financially supported by 10.13039/501100004320Shiraz University of Medical Sciences, Shiraz, Iran (Grant number: 27969). The funding body had no role in the design, data collection, analysis, interpretation of results, or writing of the manuscript.

## Authors’ contributions

All authors contributed significantly to the study design, data collection, analysis, and interpretation. They have read and approved the final manuscript.

## Conflict of interest

The authors declare that there are no conflicts of interest related to this study.
